# Toxic Leadership and Empowering Leadership: Relations with Work Motivation

**DOI:** 10.2147/PRBM.S340863

**Published:** 2022-07-28

**Authors:** Carla Santarém Semedo, Ana Salvador, Nuno Rebelo Dos Santos, Leonor Pais, Lisete Mónico

**Affiliations:** 1 Research Centre in Education and Psychology (CIEP-UÉ), Department of Psychology, School of Social Sciences, Universidade de Évora, Évora, Portugal; 2 Department of Psychology, School of Social Sciences, Universidade de Évora, Évora, Portugal; 3 Center for Research in Neuropsychology and Cognitive and Behavioral Intervention (CINEICC), Faculty of Psychology and Education Sciences, University of Coimbra, Coimbra, Portugal

**Keywords:** leadership profiles, narcissistic-empowering leaders, Empowering Leadership Questionnaire, Toxic Leadership Scale, Multidimensional Work Motivation Scale

## Abstract

**Purpose:**

The present study aims to verify the association between two opposing models of leadership, toxic and empowering, and the different dimensions of work motivation.

**Participants and Methods:**

Three questionnaires (Toxic Leadership Scale, Empowering Leadership Questionnaire, and Multidimensional Work Motivation Scale) were applied to 408 workers. Cluster analysis was performed.

**Results:**

Cluster analysis produced eight clusters based on the different configurations of leadership dimensions, namely *coercive, centralizer, false paternalistic, toxic role model, coach, narcissistic-empowering, toxic*, and *empowering*. Those clusters relate differently to work motivation dimensions.

**Conclusion:**

The profiles with higher scores in empowering leadership dimensions are more generally related to autonomous regulation (identified and intrinsic work motivation). The profiles with higher toxic leadership dimensions are more related to amotivation and controlled regulation (extrinsic work motivation). One profile stood out: the *narcissistic-empowering* profile that combines high scores in narcissistic leadership and empowering leadership dimensions. This profile unexpectedly relates significantly to autonomous regulation. This study contributed to the knowledge of the work motivation dimensions concerning empowerment and toxic leadership. Therefore, it contributes to deepening the nomological network of the concepts and providing inputs to human resource processes suitable to improving the organizational results and the workers’ quality of life.

## Introduction

The volatility and high complexity of organizational environments present demanding challenges to management. The way people are conducted in their work may lead to different outputs, which is probably one reason why leadership has been so broadly studied.

Different types of leadership in different contexts, its consequences, and its associations with other variables have mainly been studied over the last 70 years and continue to be at the core of researchers’ interest.^1–4^

While some types of leadership are defined positively, others underline the negative consequences. Two of those opposite types of leadership are empowering and toxic leaderships.^5^,^6^ The first one is defined by its positive effects on subordinates, empowering them, the second one is defined by its adverse effects undermining subordinates’ development and personal expression. Therefore, both the understanding of what behaviors make leadership empowering and what behaviors make a leader toxic are equally essential to dissemination good practices or preventing the bad ones.^7^ This study goes further concerning previous research in considering 1) the real leaders can combine in the way they perform their role both empowering and toxic leadership dimensions, displaying specific profiles regarding those dimensions; 2) those specific leadership profiles are expected to have different relationships with work-related outcomes as work motivation; 3) work motivation is approached in a complex way focusing on the several dimensions proposed by the self-determination theory; 4) leadership types are approached as social influence processes that make easier or harder to employees to obtain basic psychological needs satisfaction at work when performing their jobs.^8^,^9^

### Empowering Leadership

Considering the need to value human resources, virtuous and positive leadership models take prominence, and due to their focus on employees’ development, empowering leadership emerges in leadership literature. Empowering leadership is described as the leaders’ behaviors of sharing power towards individuals or teams by promoting their participation, autonomy, and decision-making, sharing information, delegating authority and responsibility.^10^,^11^

Such leaders’ behaviors boost meaningful work and create working conditions that increase cognitive demands and cognitive resources, leading to positive work-related feelings like employee engagement.^12–15^ Moreover, empowering leadership is different from other related constructs, such as psychological empowerment, often inaccurately mixed.^10^ This last construct can be one of the consequences of empowering leadership.

As a consequence of empowering leadership, positive psychological states are fostered in employees, influencing their motivation, their work attitudes, like proactive behaviors, and their performance.^16–19^ Performance behaviors like citizenship behavior and creativity are stimulated.^20^ The latest one is particularly evident when employees present high organizational-based self-esteem and have access to resources.^21^ It was also demonstrated that empowering leadership leads to employee’s well-being when considering some moderation variables, like perceived organizational support, perceived co-workers support, and employees’ psychological capital.^14^,^22^ The potential benefits of empowering leadership seem to be settled by those mentioned above, both for individuals and organizations. However, some contradictory results were also obtained regarding the effectiveness of empowering leadership, as shown by Cheong et al,^10^ being then relevant to clarify the relationships between that leadership type and work-related variables.

### Toxic Leadership

On the antipodes of empowering leadership, the taxonomy of negative leadership includes different concepts, such as abusive leadership, destructive leadership, and toxic leadership.^23–25^ In what concerns toxic leadership, research mainly focused on toxic leadership patterns, leaders’ traits, and their destructive behavior.^6^,^26–29^ Toxic leadership is a kind of maladjusted, malcontent and malevolent leadership by which an individual, by virtue of his destructive behavior and his dysfunctional characteristics, inflicts serious and enduring harm on the individuals, groups, organizations, and communities.^28^,^29^ There is a cumulative effect of demotivational behavior on peoples’ morale and climate over time, and the inflicted damages often continue even after the perpetrator has left the organization.^30^,^31^

Lippman-Blumen conceives a toxic leader is someone who consistently exhibits dysfunctional behavior and toxic traits as lack of integrity and honesty, egotism, and arrogance focuses on its ambition and holding power with no concern about others’ well-being.^32^ She also states their actions as intimidating, demoralizing, demeaning, and marginalizing. As a result of peoples’ needs for authority, security, belonging, feeling special, fear of ostracism, and powerlessness, they often submit to its dysfunctional way of being led.

Schmidt defines toxic leaders as narcissistic, self-promoters who engage in unpredictable abusive and authoritarian supervision patterns.^6^ Green found four patterns concerning toxic leadership: egotism (self-centered), ethical failure (lying, blaming others, abusing of power), incompetence (poor human relations skills such as listening), and neuroticism (eg, wild mood changes).^26^ Walton attempted to extend the explanation of toxic leadership to situational aspects. He pointed out the importance of paying attention to the psychological predisposition of the leader, to the internal context which allows or encourages their action, and the broader external circumstances (eg, instability) that affect the organization and may cover and excuse the dysfunctional behavior.^33^

The impact on employees and organizations of toxic leadership has been a concern. Although evidence points to adverse outcomes, Pelletier refers that, based on social construction theory, Hunt advocates that employees’ perceptions of the effectiveness or toxicity of the leader may be different according to psychological aspects of the evaluator or the established relation between leader and follower.^7^ Milosevic, Maric and Loncar highlight that in some cases, subordinates not only refuse to follow toxic leaders but they also work to minimize or neutralize their harmful impacts.^34^ Thinking differently, some workers feel that being close to the leader brings them a sense of meaning and belonging, power, and participation in decision-taking.^28^,^32^

Socio-demographic variables, like gender, also seem to play a role in perceiving leaders’ toxicity. Females perceive these leaders more negatively, probably because they tend to detailed information processing, while men present more behaviors consistent with colluders, possibly due to their heuristic processing, emphasis on positive information, or search for personal self-advantage.^35^

A leader’s toxic behavior is related to employees’ psychological distress, with adverse effects that may range from loss of self-worth, withdrawal, agitation, avoidance, worry, and holding feedback and contributions.^36^,^37^ To cope with it, problem-solving, emotional-focused strategies, or a mix of both, are used by followers.^38^ Withdrawal is associated with assertive and avoidance coping strategies to deal with the leader, while agitation is more related to avoidance and adaptive strategies. People who tend to see those behaviors as something transitional use mostly adaptive coping strategies, trying to maintain a positive relationship with the leader.^36^

Studies correlating toxic leadership to other work-related variables show various adverse effects, such as lower organizational commitment and lower job satisfaction and turnover intention among followers.^25^,^39^ Emotional disturbance, anti-social behaviors, reduced performance in employees and teams can be consequences of leader toxicity.^40^,^41^ The complexity of toxic leadership contingency relations to other variables and its consequences on employees, teams, and organizations made this topic a broad field to deepener research. Furthermore, the same leader can express a mixture of toxic and empowering leadership dimensions in their behavior, creating unique profiles. Studying the specific relationships between those unique profiles and work-related variables contributes to deepening the understanding of leadership phenomena at the workplace.

### Work Motivation

Work motivation has been one of the central topics in organizational studies.^42–44^ Deci defined motivation as a term related to three fundamental questions concerning behavior regulation: what energizes the action, how it is directed, and to what extent it is voluntarily controlled.^45^

Self-determination theory (STD) has made a turnout point in motivation study, because previous research conceived motivation in a dual quantitative perspective (people were motivated or not), as a predictor of performance and well-being.^46^,^47^ This theory identifies distinct types of motivation. It proposes a multidimensional conceptualization in a self-determination continuum (from amotivation to intrinsic motivation). SDT allows determining the motivation level (autonomous or controlled), differentiating results and the process to achieve them.^46^,^48^ Thus, besides distinguishing motivation quantitatively, it also distinguishes it qualitatively.^49^ To determine the type of motivation people are engaged in, three psychological needs must be considered: competence, autonomy, and relatedness.^46^ Those needs combine differently along the regulation continuum: amotivation (not motivated at all), extrinsic regulation (external reinforces and punishments regulate motivated behavior), introjected regulation (external motivators are introjected), identified regulation (values and the intrinsic value of the behavior’s consequence are the motivators), integrated regulation (the values and the intrinsic value of the behavior are part of the self and the value system of the subject), and intrinsic regulation (the specific behavior is a source of enjoyment and fulfilling).

From the moment STD was conceptualized, there has been a substantial proliferation of studies in different contexts framed in this approach.^48^,^50–54^

In the field of organizations, research within this framework is very relevant.^55^,^56^ Some authors pointed out that self-determined employees are more committed to the organization, report higher work satisfaction and lower emotional exhaustion, leading to fewer turnover intentions.^57^,^58^ Moreover, those employees present low daily hassles, which are negatively related to physical and stress-related symptoms.^59^ Self-determination theory advocates that the distinct types of motivation, from more externally controlled to more autonomous, impact differently and have specifiable consequences on learning, performance, personal experience, and well-being.^46^ Moreover, being those different work motivation types psychologically different, we can expect their determinants are also different.

### Empowering Leadership, Toxic Leadership and Work Motivation

Literature relating leadership to work motivation is scarce and looks at motivation as a dichotomous variable: intrinsic or extrinsic, not considering its degree of internalization.^16^,^60–62^ Studies are even more scarce when seeking associations between specifically empowering leadership, toxic leadership, and self-determination theory. Chen, Sharma, Edinger, Shapiro, and Farh demonstrated the motivator effect of empowering leadership and the demotivating effect of conflicting relationships in teams by verifying the role of team members’ motivational states in innovation, teamwork, and turnover intention.^63^ However, the three dimensions of motivation conceived by Ryan and Deci within SDT were not considered, and the assumption of its different effects was not studied.^46^ Syahrul also found a significant effect of empowering leadership on intrinsic motivation, but no other types of work motivation were considered.^62^

Regarding leader autonomy support, the meta-analysis undertaken by Slemp et al confirms an overall pattern that this characteristic of leadership is strongly related to autonomous regulation at work.^9^ That concept (leader autonomy support) is close to empowering leadership. However, those authors do not include other possible complex combinations of empowering leadership dimensions with other aspects of how leadership role is performed.

In what concerns toxic leadership, as far as we know, no empirical study has been carried out linking this type of leadership to work motivation. Reed refers to its cumulative demotivational effect on teams, but that argument is conceptual.^30^ Moreover, the five dimensions of each of these two antagonistic types of leadership are not jointly analyzed in the studies we have mentioned above. Real leaders can perform their roles by mixing behaviors in unique profiles of toxic and empowering leadership dimensions.

Given the revised literature and the identified gaps, along with the complexity of the discussed variables, it seems reasonable to conceive that different leadership profiles combining toxic and empowering leadership components can produce distinct patterns in what concerns its association with work motivation. Therefore, the objective of our study is to characterize different leadership profiles and analyze their differential association with the various dimensions of work motivation. Since this is not a hypotheses test study, our research questions are as follows: 1) To what extent do different leadership profiles emerge (based on empowering and toxic leadership dimensions)? and 2) What are the significant associations between those leadership profiles and work motivation dimensions (according to the self-determination theory)?

## Materials and Methods

### Participants and Procedure

Students from a public university enrolled in a psychology degree were trained to collect data within the scope of a course on research methodology. They learned rigorous methodological procedures and ethical standards concerning data collection. Eventually, each student was appointed to collect responses from 5 to 10 participants and made a written report of the process. They collected data from acquaints or relatives complying with the eligibility criteria: 1) Participants must be employees belonging to public or private organizations larger than nine workers; 2) Working there for at least 3 months with the same leader. That last condition aimed to assure they had a clear opinion about the leadership characteristics and their own work motivation. After data collection, each student wrote a report describing the process and included the information regarding questions or comments from respondents and the corresponding responses provided. The students also signed a responsibility statement concerning compliance with the data collection procedure.

A sample of 408 participants was collected (233 women), ranging from 18 to 76 years old. Regarding academic qualifications, 27.5% had qualifications until primary education, 38% finished secondary education, and 34.1% had higher education (0.4% missing values). In addition, 76.2% of the participants reported not having a leadership role within the organization. Regarding the organizational size, 16.9% had up to 9 employees, 31.6% from 10 to 50, 22.1% from 51 to 250 employees, and the remaining above 250 employees.

The study was conducted in accordance with the Declaration of Helsinki. Furthermore, all the formal and ethical requirements of the psychological research were met, including approval from the ethical committee of the public university. Before responding to the questionnaire, participants received information regarding the general objectives of the study and assurance of anonymity and confidentiality of the data. After participation acceptance, they were asked to sign the written informed consent. This document was presented in a separate sheet, later kept by the principal investigator.

### Measures

Participants responded to three instruments:

1) Toxic Leadership Scale (TLS), adapted to a Portuguese sample by Mónico et al, composed of 30 Likert-type items, organized in five dimensions: Abusive Leadership (eg, “Speaks poorly about subordinates to other people in the workplace”), Authoritarian Leadership (eg, “Controls how subordinates complete their tasks”), Narcissism (eg, “Has a sense of personal entitlement”), Self-promotion (eg, “Accepts credit for successes that do not belong to him/her”), and Unpredictability (eg, “Has explosive outbursts”)^5^,^6^;

2) Empowering Leadership Questionnaire (ELQ) also validated by Mónico et al in a sample of Portuguese workers, is composed of 38 Likert-type items, in five dimensions: Leading by example (eg, “Sets high standards for performance by his/her own behavior”), Participative Decision making (eg, “Encourages work group members to express ideas/suggestions”), Coaching (eg, “Helps my work group see areas in which we need more training”), Informing (eg, “Explains company decisions”), and Showing concern (eg, “Cares about work group members’ personal problems”)^5^,^64^;

3) Multidimensional Work Motivation Scale (MWMS), validated in Portuguese and Brazilian samples of workers by Dos Santos et al, composed of 18 items in six dimensions: Amotivation (eg, “I don’t know why I’m doing this job, it’s pointless work”), Extrinsic Regulation (material and social, (eg, “Because I risk losing my job if I don’t put enough effort in it” (material)); “To avoid being criticized by others (eg, supervisor, colleagues, family, clients … ” (social)), Introjected Regulation (eg, “Because I have to prove to myself that I can”), Identified Regulation (eg, “Because I personally consider it important to put efforts in this job”) and Intrinsic Motivation (eg, “Because I have fun doing my job”).^48^,^65^ Additionally, sociodemographic questions were included.

The scales’ dimensions showed excellent reliability (see Table 1, Cronbach’s alphas from 0.812 to 0.962). Composite reliability was also good since higher than 0.70.^66^

**Table 1:** Descriptives (M;SD), Composite Reliability (CR), Average Extracted Variance (AVE), Cronbach’s Alphas (Between Brackets), and Correlation Matrix Between Multidimensional Work Motivation Scale (MWMS), Empowering Leadership Questionnaire (ELQ) and Toxic Leadership Scale (TLS) [Squared Correlations R^2^ Between Square Brackets]

	M	SD	CR	AVE	(1)	(2)	(3)	(4)	(5)	(6)	(7)	(8)	(9)	(10)	(11)	(12)	(13)	(14)	(15)	(16)
MWMS F1 (1)	1.71	1.24	0.910	0.770	(0.905)	0.323** [0.104]	0.183* [0.033]	0.044 [0.002]	−0.214** [0.046]	−0.173** [0.030]	−0.111*	−0.138**	−0.113*	−0.143**	−0.121*	0.179**	0.142**	0.090	0.129**	0.121*
MWMS F2.1 (2)	3.74	1.81	0.880	0.710		(0.879)	0.477**	0.383**	0.119*	0.071	0.121*	0.128**	0.172**	0.129**	0.135**	0.002	−0.033	0.005	0.010	0.042
MWMS F2.2 (3)	2.87	1.81	0.830	0.620			(0.812)	0.423**	0.122*	0.004	0.121*	0.063	0.156**	0.164**	0.104*	0.065	0.079	0.037	0.035	0.115*
MWMS F3 (4)	4.56	1.72	0.760	0.440				(0.830)	0.578**	0.376**	0.199**	0.192**	0.192**	0.242**	0.189**	0.013	−0.007	−0.015	−0.027	0.044
MWMS F4 (5)	5.61	1.52	0.730	0.470					(0.909)	0.702**	0.339**	0.330**	0.337**	0.352**	0.357**	−0.210**	−0.217**	−0.186**	−0.241**	−0.214**
MWMS F5 (6)	4.85	1.72	0.750	0.500						(0.931)	0.351**	0.372**	0.381**	0.389**	0.383**	−0.214**	−0.203**	−0.163**	−0.228**	−0.233**
ELQ F1 (7)	3.45	1.07	0.910	0.670							(0.906)	0.706** [0.498]	0.772** [0.596]	0.637** [0.406]	0.718** [0.516]					
ELQ F2 (8)	3.30	0.96	0.910	0.640								(0.908)	0.838**	0.677**	0.822**					
ELQ F3 (9)	3.35	1.01	0.960	0.700									(0.962)	0.803**	0.882**					
ELQ F4 (10)	2.73	1.34	0.940	0.730										(0.942)	0.768**					
ELQ F5 (11)	3.46	0.98	0.950	0.660											(0.949)					
TLS F1 (12)	2.28	1.21	0.930	0.640												(0.873)	0.777** [0.604]	0.720** [0.518]	0.722** [0.521]	0.752** [0.566]
TLS F2 (13)	2.65	1.16	0.880	0.550													(0.923)	0.770**	0.731**	0.719**
TLS F3 (14)	3.39	1.05	0.910	0.680														(0.913)	0.824**	0.693**
TLS F4 (15)	2.49	1.33	0.910	0.670															(0.907)	0.732**
TLS F5 (16)	2.72	1.40	0.950	0.720																(0.946)

**Notes**: ***p ≤ 0.001, **p <0.01, *p <0.05.

**Abbreviations**: M, mean; SD, standard-deviations; MWMS F1, MWMS Amotivation; MWMS F2.1, MWMS Material External Regulation; MWMS F2.2, MWMS Social External Regulation; MWMS F3, MWMS Introjected Regulation; MWMS F4, MWMS Identified Regulation; MWMS F5, MWMS Intrinsic Motivation; ELQ F1, ELQ Leading by example; ELQ F2, ELQ Participative Decision-making; ELQ F3, ELQ Coaching; ELQ F4, ELQ Informing; ELQ F5, ELQ Show concern; TLS F1, TLS Abusive Leadership; TLS F2, TLS Authoritarian Leadership; TLS F3, TLS Narcissism; TLS F4, TLS Self-promotion; TLS F5, TLS Unpredictability.

We found an acceptable convergent validity between the items of each factor for the three measures, concerning the average variance extracted (AVE) scores: Factors F3 and F4 of the MWMS exceed the cut-off value of 0.4078 and the remaining factors of all the scales exceed 0.50, ensuring that the explained variance is greater than the residual variance.^67^ Regarding discriminant validity, the average variance extracted from each factor is greater than the values of the squared correlations between each pair of factors (R^2^) for each scale^68^ (see Table 1, squared correlations R^2^ between square brackets).

### Data Analysis

Data was processed with IBM SPSS Software (IBM Corp. Released 2013). Missing-values, all MCAR (completely random), and less than 2% were replaced by the Series mean method.

The normality of the variables was assessed by the skewness (*sk*) and kurtosis (*ku*) coefficients. We obtained scores of | *sk* | < 2 and |*ku*| < 6. Outliers were analyzed according to the Mahalanobis squared distance,^69^ with no relevant values being found, having all data been considered.

Reliability was calculated by Cronbach’s alpha.^70^ The score of 0.80 was taken as a good reliability indicator.^71^ The composite reliability and the average variance extracted for each factor were evaluated for convergent and discriminant validity as described by Schumacker and Lomax.^72^ Associations between factors were performed through Pearson correlations, being effect sizes of correlations classified according to Cohen.^73^

After descriptive statistics and intercorrelation matrix, a cluster analysis was performed to identify leadership profiles. The TwoStep method for continuous variables is a scalable cluster analysis algorithm, which defines the ideal number of clusters.^74^ The distance measure was calculated with the Log-Likelihood distance measurement. Schwarz’s Bayesian Criterion was used for the classification of clusters, which indicated for each dimension of ELQ and TLS the number of clusters with good quality of discrimination (silhouette measure of cohesion and separation >0.50).

After creating the leadership profiles, differences regarding MWMS scores were analyzed through the Mann–Whitney non-parametric test, considering the distribution of the sample was not normal in most profiles (Shapiro–Wilk normality tests were performed) and the variances were heterogeneous.

A significance level of 0.05 for Type I error was considered for all the analyses.

## Results

### Descriptive Analysis and Correlations

MWMS, ELQ and TLS showed a good internal consistency (see Table 1, Cronbach’s alphas). The factor with the highest mean value was *Identified Regulation* and the lowest was *Amotivation* (see Table 1 for means and standard-deviations). In the empowerment leadership, the factor that showed the highest average value was *Showing Concern* and the lowest was *Informing*. In toxic leadership, the factor that showed the highest average value was *Narcissism* and the lowest was *Abusive Supervision*. MWMS correlated positively with ELQ in the vast majority of factors, except for the *Amotivation* dimension, with which it correlated negatively. Regarding the TLS, it reveals a negative correlation with the dimensions *Identified Regulation* and *Intrinsic motivation*, correlating positively with the *Amotivation*.

Regarding the dimensions of the ELQ, we can see that as we move towards the dimensions of more autonomous motivation, the higher the correlations are. We found weak negative correlations between all factors of the ELQ and *Amotivation*. With regard to the correlation between ELQ factors and *Material External Regulation, Social External Regulation*, and *Introjected Regulation*, we can verify the existence of weak positive correlations.^68^ It is with the *Identified Regulation* and *Intrinsic Motivation* that all ELQ factors correlated moderately.^68^ Among the ELQ factors, those that correlate most highly with the MWMS dimensions were *Coaching, Informing*, and *Showing concern*. We found that all dimensions of ELQ are related with work motivation, and this relationship is more evident with *Intrinsic Motivation*.

Regarding the TLS, we verified the greatest number of correlations not statistically significant, namely with the dimensions *Social* and *Material External Regulation* and *Introjected motivation*. In addition, TLS correlated weakly with *Amotivation* (positive correlation) and with *Identified Regulation* and *Intrinsic Motivation* (negative correlations). Among the TLS factors with the highest negative correlations with the MWMS dimensions were *Abusive Leadership, Self-promotion*, and *Unpredictability*.

### Cluster Analysis

Each dimension of the ELQ and TLS was individually submitted to cluster analysis (log-likelihood distance measurement, Schwarz Bayesian criterion), aggregated in two (low and high) and three (low, intermediate, and high) clusters. All clusters showed good quality of discrimination (see Table 2).

**Table 2:** Number of Clusters, Quality of Discrimination, Number of Participants in Each Cluster (%, n) and Means (M) for the Dimensions of the ELQ and TLS

Cluster	Number of Clusters	Quality of Discrimination	Low		Intermediate		High	
			% (n)	M	% (n)	M	% (n)	M
ELQ F1—Leading by example	2	0.7	44.6 (182)	1.21	-	-	55.4 (226)	3.89
ELQ F2—Participative Decision-making	3	0.7	23 (94)	1.94	42.9 (175)	3.21	34.1 (139)	4.31
ELQ F3—Coaching	2	0.7	31.1 (127)	2.11	-	-	68.9 (281)	3.92
ELQ F4—Informing	2	0.7	50.5 (206)	2.52	-	-	49.5 (202)	4.19
ELQ F5—Show concern	3	0.7	33.6 (137)	1.67	25.2 (103)	3.42	41.2 (168)	4.81
TLS F1—Abusive Leadership	3	0.7	48.8 (199)	1.30	31.1 (127)	2.52	20.1 (82)	4.25
TLS F2—Authoritarian Leadership	2	0.7	62.7 (256)	1.91	-	-	37.3 (152)	3.88
TLS F3—Narcissism	3	0.7	21.3 (87)	1.61	35.3 (144)	3.37	43.4 (177)	5.14
TLS F4—Self-promotion	2	0.7	63. (257)	1.62	-	-	37 (151)	3.97
TLS F5—Unpredictability	2	0.7	66.7 (272)	1.88	-	-	33.3 (136)	4.41

After obtaining the clusters in each dimension of the ELQ and the TLS, we found that the participants belonged to different leadership clusters (eg, high versus low/medium) in different dimensions of the same questionnaire. Thus, joining the participants belonging to each cluster in the five dimensions of ELQ and TLS led to the emergence of eight different leadership profiles, described in Table 3. Based on Goleman’s classification we considered and named the following worker leadership profiles: 1) Coercive, requiring immediate obedience; 2) Centralizer, the leader usually takes decisions, subsequently informing all the steps to be followed; 3) False paternalistic, building emotional bonds, although exercising abusive and unpredictable supervision; 4) Toxic role model, emphasizing high-performance standards, however in a toxic way; () Coach, who develops people, however, only following his own rules; 6) Narcissistic-empowering, who recognizes his or her collaborators but only in favoritism of the self-interests; 7) Toxic, exercising a nefarious leadership; and finally, 8) Empowering, revealing a beneficial leadership style.^75^,^76^ For example, the coercive profile concerns individuals with high scores in ELQF2 (Participative Decision-making), TLSF3 (Narcissism) and TLSF4 (Self-promotion), and medium/low scores in the remaining dimensions, which corresponds to individuals who demand immediate obedience from their subordinates, meaning that their own opinions are superior, despite sharing challenges.^75^,^76^

**Table 3:** Profiles Creation in Function of the Belonging to the High vs Low/Medium Cluster in Each Dimension of ELQ and TLS

Profile	n	ELQ F1	ELQ F2	ELQ F3	ELQ F4	ELQ F5	TLS F1	TLS F2	TLS F3	TLS F4	TLS F5
1 .Coercive	14	Low/medium	High	Low/medium	Low/medium	Low/medium	Low/medium	Low/medium	High	High	Low/medium
2 .Centralizer	25	Low/medium	Low/medium	Low/medium	High	Low/medium	Low/medium	High	Low/medium	Low/medium	Low/medium
3. False paternalistic	20	Low/medium	Low/medium	Low/medium	Low/medium	High	High	Low/medium	Low/medium	Low/medium	High
4. Toxic role model	15	High	Low/medium	Low/medium	Low/medium	Low/medium	Low/medium	High	High	High	Low/medium
5. Coach	21	High	Low/medium	Low/medium	High	High	Low/medium	High	Low/medium	Low/medium	Low/medium
6. Narcissistic- empowering	118	High	High	High	High	High	Low/medium	Low/medium	High	Low/medium	Low/medium
7. Toxic	45	Low/medium	Low/medium	Low/medium	Low/medium	Low/medium	High	High	High	High	High
8. Empowering	28	High	High	High	High	High	Low/medium	Low/medium	Low/medium	Low/medium	Low/medium

**Abbreviations**: ELQ F1, Leading by example; ELQ F2, Participative Decision-making; ELQ F3, Coaching; ELQ F4, Informing; ELQ F5 Show concern; TLS F1, Abusive Leadership; TLS F2, Authoritarian Leadership; TLS F3, Narcissism; TLS F4, Self-promotion; TLS F5, Unpredictability.

The worker leadership profile with the highest number of participants was the *narcissistic-empowering* (n = 118), followed by the *toxic* profile (n = 45). Conversely, the profiles with the lowest number of participants were the *coercive* (n = 14) and the *toxic role model* (n = 15). We proceed with the analysis of differences between worker leadership profiles in terms of MWMS dimensions. First, we performed the Mann–Whitney non-parametric test, using as independent variables (IV) the pertaining or not to each profile (1 = yes; 0 = no) and as dependent variables (DV) the six dimensions of the MWMS (see Table 4).

**Table 4:** Means (M), Standard Deviations (SD), Mean-Rank (MR) and Mann–Whitney *U*-Tests for the Eight Leadership Worker Profiles of Workers in Each Dimension of Work Motivation

	DV	Amotivation				Material External Regulation			Social External Regulation			Introjected Regulation			Identified Regulation			Intrinsic Motivation		
Profile		n	*M*	*SD*	*MR*	*M*	*SD*	*MR*	*M*	*SD*	*MR*	*M*	*SD*	*MR*	*M*	*SD*	*MR*	*M*	*SD*	*MR*
1. Coercive	No	394	1.7	1.2	203.6	2.8	1.8	201.7	3.7	1.8	201.6	4.5	1.7	201.8	5.6	1.5	204.2	4.8	1.7	204.4
	Yes	14	2.2	1.7	231.2	4.3	2.1	283.3	4.9	1.6	285.5	5.6	1.4	278.7	5.9	1.1	212.3	4.9	1.8	206.4
*Mann–Whitney U*		2384.0				1654.0			16230.5			1719.5			2649.5			2732.0		
*Z*		−0.97				−2.58*			−2.62**			−0.25*			−0.25			−0.06		
2. Centralizer	No	383	1.7	1.2	199.9	2.9	1.8	204.9	3.8	1.8	206.2	4.5	1.7	206.3	5.7	1.5	209.6	4.9	1.7	209.9
	Yes	25	2.2	1.1	274.6	2.7	1.6	198	3.3	1.4	178.6	4.2	1.6	177.7	4.6	1.7	127.1	3.7	1.6	122.3
*Mann–Whitney U*		3034.0				4625.0			4139.0			4118.0			2853.5			2733.0		
*Z*		−3.46**				−0.29			−1.14			−1.17			−3.44**			−3.61***		
3. False paternalistic	No	388	1.7	1.3	204.4	2.9	1.8	203.7	3.7	1.8	202.8	4.5	1.7	202.6	5.6	1.5	204.6	4.9	1.7	206.8
	Yes	20	1.7	1.1	206.8	3.2	2.0	219.5	4.3	1.8	238.5	5.1	1.5	241.6	5.6	1.4	202.4	4.3	1.5	159.5
*Mann–Whitney U*		3833.5				3581.0			3200.5			3137.5			3837.0			2980.5		
*Z*		−0.10				−0.58			−1.32			−1.45			−0.09			−1.76		
4. Toxic role model	No	393	1.7	1.6	203.3	2.9	1.8	205.0	3.7	1.8	203.6	4.6	1.7	204.2	5.6	1.5	205.5	4.9	1.7	205.2
	Yes	15	1.7	0.8	236.2	2.7	1.9	190.9	4.1	1.6	227.5	4.	1.7	212.0	5.4	1.4	178.2	4.6	1.8	186.2
*Mann–Whitney U*		2471.5				2743.5			2602.5			2835.0			2554.0			2672.5		
*Z*		−1.20				−0.46			−0.77			−0.25			−0.89			−0.62		
5. Coach	No	387	1.7	1.2	203.2	2.8	1.8	201.1	3.7	1.8	202.2	4.5	1.7	202.1	5.6	1.5	204.5	4.8	1.7	203.9
	Yes	21	1.9	1.3	228.0	3.9	2.0	268.2	4.4	1.5	247.7	5.2	1.5	248.8	5.7	1.4	205.4	4.9	1.7	214.2
*Mann–Whitney U*		3568.5				2726.5			3157.0			3133.5			4044.0			3859.0		
*Z*		−1.06				−2.57*			−1.73			−1.77			−0.038			−0.39		
6. Narcissistic- empowering	No	290	1.8	1.2	218.1	2.9	1.8	206.2	3.8	1.8	205.8	3.8	1.8	197.0	5.3	1.6	184.2	4.5	1.8	183.9
	Yes	118	1.6	1.4	171.03	2.9	1.9	200.4	3.7	2.0	201.2	4.8	1.8	222.9	6.2	1.1	254.3	5.6	1.3	255.2
*Mann–Whitney U*		13160.0				16630.5			16754.5			14942.5			11234.0			11124.5		
*Z*		−4.13***				−0.45			−0.36			−2.01*			−5.53***			−5.56***		
7. Toxic	No	363	1.7	1.3	202.9	2.9	1.8	208.5	3.8	1.8	206.5	4.6	1.7	207.2	5.7	1.5	206.9	4.9	1.7	206.8
	Yes	45	1.7	0.9	217.2	2.3	1.4	172.2	3.5	1.9	188.8	4.2	1.8	182.8	5.3	1.8	184.7	4.6	1.9	186.0
*Mann–Whitney U*		7597.5				6714.0			7460.0			7139.0			7276.5			7337.0		
*Z*		−0.86				−1.97*			−0.95			−1.31			−1.21			−1.12		
8. Empowering	No	382	1.7	1.3	206.4	2.9	1.8	205.9	3.8	1.8	205.4	4.5	1.7	202.7	5.6	1.5	202.0	4.8	1.7	200.9
	Yes	26	1.4	0.9	177.1	2.5	1.5	184.1	3.5	1.6	191.1	4.9	1.4	230.6	6.2	0.9	241.3	5.6	1.2	257.6
*Mann–Whitney U*		4252.5				4435.0			4618.5			4287.5			4009.5			3586.5		
*Z*		−1.35				−0.92			−0.59			−1.17			−1.67			−2.38*		

**Notes**: *p < 0.05; **p < 0.01; ***p < 0.001.

We found that workers from our sample have different leadership profiles— some of those profiles distinguished in several dimensions of work motivation. The coercive, centralizer and narcissistic-empowering profiles presented significant differences in three dimensions of work motivation, while coach, toxic, and empowering profiles showed significant differences only in one dimension. The remaining profiles (false paternalistic and toxic role model) did not show significant differences in any work motivation dimension.

As shown in Table 4, participants who belong to the *coercive* leadership profile had significantly higher means in *External Regulation* (material and social) and *Introjected Regulation* compared with participants not included in that profile. The *Material External Regulation* dimension of work motivation was significantly higher in participants within the *coach* profile and significantly lower in participants within the *toxic* profile. *Intrinsic motivation* and *Identified Regulation* were higher in participants within the *narcissistic-empowering* profile than participants from other leadership profiles. Inversely, *Intrinsic motivation* and *Identified Regulation* presented lower scores in participants within the *centralizer* profile than participants from other profiles. Considering *Amotivation*, the participants of the *narcissistic-empowering* profile score lower, and those of the *coercive* profile score higher.

## Discussion

This research aimed to characterize different leadership profiles and analyze their differential association with the dimensions of work motivation. To do that, we performed cluster analysis. Clustering was based on the *toxic* and *empowering* leadership dimensions and then related to differences in work motivation among clusters. We performed a cluster analysis to test specific leadership profiles and how those relate to the subordinates’ work motivation. This approach was used by Schilling and Schyns in their study about causes and consequences of bad leadership.^77^

Through this method, we were able to identify eight different profiles in our sample. Following Goleman’s classification, we named them *coercive, centralizing, false paternalistic, toxic role model, coach, narcissistic-empowering, toxic*, and *empowering*.^75^,^76^ The *false paternalistic* and the *toxic role model* are indistinct in their relation to the subordinates’ work motivation.

The *coercive* profile is distinguishable in *extrinsic* (both *material* and *social*) and *introjected* dimensions of motivation. This profile is the one in which the leader demands immediate obedience, not allowing interventions from workers. The leader frightens people and is moved by results only. For this reason, he or she presents many characteristics from the toxic leader, using punishing strategies and negative feedback to reach his or her goals. Additionally, that leader mobilizes workers by a motivation to succeed, initiative, and self-control.^76^ Therefore, it seems plausible that subordinates feel demotivated since they are pressed to task accomplishment having no autonomy or creativity, making them feel tense in the face of the possibility of a mistake and retaliation. Accordingly, employees may be more exposed to psychological illness in this type of leadership, like stress and depression.^36^,^78^ However, that does not mean they become necessarily ill. Instead, they can use the consequences of working to obtain basic psychological needs satisfaction outside the workplace and reinforce their extrinsic work motivation. Where subordinates have a coercive leader, they do not feel they get psychological needs satisfaction from working, and controlled regulation dominates their drive to work.^8^

The *centralizer* profile is distinguished by more demotivated followers (amotivation) having less identified and intrinsic motivation than others. This is a profile where the leader guides subordinate to what they should do and how they should act. He or she plans and coordinates work, sets standards, centralizes power on him or herself, and does not ask for opinions. This profile encompasses characteristics of a toxic and abusive leader on the one hand and an enabler/supporter, on the other hand, mobilizing individuals through a vision, transmitting self-confidence and empathy differing, thus, from the previous profile.^76^ An explanatory hypothesis for this profile association is a lower work identification (since the worker plays only the operational component). The leader appropriates him or herself of the work and creativity, leaving the subordinate unmotivated for the tasks. Even though the leader provides a vision, this profile may create meaningless work for those who undertake the tasks due to the lack of agency and competence need satisfaction.^8^

The *coach* profile, associated in our sample to a more extrinsic material motivation, is characterized by the ability to identify highly quickly strengths and weaknesses in his or her team, being patient, humble, respectful, and success-oriented. This leader does not give up on subordinates when they have difficulties at work, encouraging and mobilizing them through the leader’s initiative, motivation for success, and also for his or her discipline. This profile encompasses characteristics of an empowering leader, showing commitment to the team and giving them autonomy and independence, although exceeding her or himself sometimes in terms of discipline.^76^ It is not easy to understand why this profile differentiates participants who belong to it from those who do not only by the amount of material extrinsic motivation. Other differences would be expected. One possible explanation is that the leader stimulates goal-oriented work regarding financial outcomes. However, future studies may help to understand this result, mainly because only 21 participants belong to this profile in our sample.

The *narcissistic-empowering* profile is by far the one that includes more participants (N = 118). This pattern is lower in amotivation and is higher in introjected, identified, and intrinsic motivation. Narcissistic-empowering leaders are negatively described in the literature as governed by vanity, exhibitionism, and empathetic incapability, showing at the same time a sense of superiority.^79^ However, in our investigation, these narcissistic-related attributes aggregate to the empowering leader characteristics in those belonging to this profile. In fact, in our sample, leadership within this cluster scores high in all empowering dimensions, while the toxic leadership profile no. Thus, this leader will be expected to inspire his or her team when subordinates have common interests, mobilizing them through his or her motivation to succeed, initiative, and self-control.^76^ Additionally, self-evaluation, the difficulty in accepting others’ errors, his or her need to stand out and the lack of empathy may lead to a destructive effect on subordinates primarily and also in the organization itself.^80^ This does not seem to happen in our sample. On the contrary, the narcissistic dimension seems not affect negatively the intrinsic, identified and introjected motivation. The leaders’ narcissism appears to be well tolerated by subordinates if high scores in all empowering dimensions are associated. Since the leader empowers subordinates, they satisfy their basic psychological needs^8^ by working and may feel the narcissistic character of the leader as something that sparks them at the workplace. The high number of participants within this profile leads us to hypothesize the presence of cultural specificity. Future research may help to clarify this question.

The *toxic* profile within this sample is associated with the most material extrinsic motivation compared to other profiles. It seems plausible that these workers get their work compensations in that type of extrinsic motivation since the remaining potential benefits are contaminated by leadership toxicity. Maybe they remain working at that work due to a lack of other opportunities to get the material earnings they need to live. Interestingly, both coach and toxic profiles seem to promote extrinsic material motivation. While the first one probably makes it by encouraging employees to set financial goals for their development, the second one by eliminating intrinsic drivers at the workplace.

Finally, the *empowerment* profile is associated in our sample with the highest *intrinsic motivation*. This profile is characterized by serving as an example to developing individuals through empathy and self-awareness. This result strengthens the conviction that *empowering* leadership tends to make work more pleasurable, as it corresponds to job enrichment. Moreover, empowering leadership makes the tasks meaningful, and the basic psychological needs satisfaction^8^ to occur at the workplace. Specifically concerning the competence need, workers experience agency and skills development; regarding the relatedness need, workers feel respected and deserving attention and consideration; and concerning autonomy need, workers participate in decision-making and have a voice.

The above analysis allowed us to verify complex leadership profiles in our sample that distinctly associate with subordinates’ work motivation dimensions. The *narcissistic* dimension of *toxic* leadership seems to be tolerated since *empowering* dimensions are also present in the exercise of leadership.

The relevance of this investigation is based mainly on the consideration that it brings an essential contribution to the design of strategies and practices of people’s management in organizations. Our results confirm that the way people are driven to work depends largely on the leadership type they have to face. Moreover, empowering leadership seems to stimulate higher work motivation forms by satisfying basic psychological needs of relatedness, competence, and autonomy. This confirms previous results showing leader autonomy support strengthens higher forms of work motivation such as identified and intrinsic work motivation.^9^ However, in our results, the effect of empowering leadership is preserved even if the leader has some narcissistic dimension in the way he or she performs the role. Furthermore, toxic, centralizer and coercive profiles should be prevented and replaced since they damage the workers’ work motivation.

In sum, our results state that the way people perceive the leader is related to their work motivation. This relationship reaffirms the importance of changes in people management policies and practices, namely in the sub-processes of recruiting, selecting, integrating, and developing leaders, as well as in the metrics used to measure performance, with a view to good management, that Maslow would consider more *eupsychian*.^81^ Organizations can thus use this study to evaluate themselves, internally analyzing the leadership profiles practiced, considering the improvement of their employees’ quality of life and the organization’s results.

We thus hope that the study of the dimensions and conceptual models covered will benefit organizational entities and individual workers. Healthy workplaces are those where individuals contribute to their work by being involved in a strong way (giving meaning to work) and improving the results of that work.

## Conclusion

This investigation aimed to understand to what extent different leadership profiles emerge (based on empowering and toxic leadership dimensions) and the significant associations between these leadership profiles and the dimensions of work motivation according to the self-determination theory.

We identified eight leadership profiles in the present study that complexly combined *toxic* leadership and *empowerment* leadership components. One profile stood out: the *narcissistic-empowering* profile that characterizes the leader as being governed by vanity, exhibitionism, empathic incapacity, and where the feeling of superiority gravitates. Additionally, he or she presents characteristics of an enabling and empowering leader, inspiring the team when subordinates have common interests, mobilizing them through their motivation for success, initiative, and self-control. One possible explanation for the positive effect of this profile on higher forms of employees’ work motivation is that the developmental strength of empowering dimensions reconfigures the meaning of narcissistic dimensions. Therefore, each dimension has no meaning per se, but its meaning depends on the end-up configuration of all leadership dimensions. So, the power of needs satisfaction of how leadership is performed is a complex process that is better understood if considered in its whole design. The way leadership is performed can make it easier or harder for employees to satisfy the basic psychological needs of autonomy, competence, and relatedness by working. Accordingly, those employees will experience different degrees of internalization of work motivation.

This study is relevant in demonstrating the importance of considering work motivation as a regulation continuum (and not a dichotomous phenomenon). The satisfaction of the competence, autonomy and relatedness needs can be facilitated or hindered by different leadership profiles. This finding is relevant to human resources practices that aim to promote the well-being of employees in organizations.

In a more conceptual perspective, it affords the knowledge of the work motivation dimensions concerning the factors of empowerment leadership and toxic leadership, contributing thus, to the deepening of the nomological network of the involved concepts.

### Limitations and Future Research

The cross-sectional design of this study does not allow to capture the evolution in the associations between the variables. It would be interesting to undertake a longitudinal study about *empowerment* and *toxic* leadership’s effect on the work motivation of subordinates.

The sampling technique applied for collecting data does not allow high generalizability. In further studies, more robust sampling techniques can verify to what extent the results obtained here are culture-specific or express more transcultural phenomena.

The effects of sociodemographic variables also can be considered in future studies. Likewise, the role of different subordinates’ characteristics as moderators of leadership style can be a relevant topic of research.^82^

Enlightening the effect of the leaders’ characteristics (eg, personality, positive and negative work experiences) in adopting an empowerment style, or a toxic style would be a matter of high interest.

Finally, it would be helpful to consider the organization characteristics (eg, strategy, supervision, company values) as mediators of the effect of leadership style on employees’ work motivation.

## References

[1] Cummings GG, Tate K, Lee S, et al. Leadership styles and outcome patterns for the nursing workforce and work environment: a systematic review. *Int J Nurs Stud*. 2018;85:19–60. doi:10.1016/j.ijnurstu.2018.04.016 29807190

[2] Dunst CJ, Bruder MB, Hamby DH, Howse R, Wilkie H. Meta-analysis of the relationships between different leadership practices and organizational, teaming, leader, and employee outcomes. *J Int Educ Leadersh*. 2018;8(2):1–45.

[3] Eva N, Robin M, Sendjaya S, van Dierendonck D, Liden RC. Servant leadership: a systematic review and call for future research. *Leadersh Q*. 2019;30(1):111–132. doi:10.1016/j.leaqua.2018.07.004

[4] Hughes DJ, Lee A, Tian AW, Newman A, Legood A. Leadership, creativity, and innovation: a critical review and practical recommendations. *Leadersh Q*. 2018;29(5):549–569.

[5] Mónico L, Salvador A, Dos Santos NR, Pais L, Semedo C. Lideranças tóxica e empoderadora: estudo de validação de medidas em amostra Portuguesa [Toxic and empowering leadership: measures validation study in a portuguese sample]. *Rev Iberoam Diagn Eval*. 2019;53(4):129–140.

[6] Schmidt A. Development and validation of the toxic leadership scale [dissertation]. Maryland: University of Maryland; 2008. Available from: https://drum.lib.umd.edu/handle/1903/8176. Accessed September 28, 2021.

[7] Pelletier K. Leader toxicity: an empirical investigation of toxic behavior and rhetoric. *Leadersh*. 2010;6(4):373–389. doi:10.1177/1742715010379308

[8] Vansteenkiste M, Ryan RM, Soenens B. Basic psychological need theory: advancements, critical themes, and future directions. *Motiv Emot*. 2020;44(1):1–31.

[9] Slemp GR, Kern ML, Patrick KJ, Ryan RM. Leader autonomy support in the workplace: a meta-analytic review. *Motiv Emot*. 2018;42:706–724. doi:10.1007/s11031-018-9698-y 30237648 PMC6133074

[10] Cheong M, Yammarino FJ, Dionne SD, Spain SM, Tsai CY. A review of effectiveness of empowering leadership. *Leadersh Q*. 2019;30:34–58. doi:10.1016/j.leaqua.2018.08.005

[11] Sharma PN, Kirkman BL. Leveraging leaders: a literature review and future lines of inquiry for empowering leadership research. *Group Organ Manag*. 2015;40(2):193–237. doi:10.1177/1059601115574906

[12] Kim M, Beehr TA. Organization-based self-esteem and meaningful work mediate effects of empowering leadership on employee behaviors and well-being. *J Leadersh Organ Stud*. 2018;25(4):385–398. doi:10.1177/1548051818762337

[13] Lee MCC, Idris MA, Delfabbro PH. The linkages between hierarchical culture and empowering leadership and their effects on employees’ work engagement: work meaningfulness as a mediator. *Int J Stress Manage*. 2017;24(4):392–415. doi:10.1037/str0000043

[14] Park JG, Kim JS, Yoon SW, Joo YB-K. The effects of empowering leadership on psychological well-being and job engagement. *Leadersh Organ Dev J*. 2017;38(3):350–367. doi:10.1108/LODJ-08-2015-0182

[15] Tuckey MR, Bakker AB, Dollard MF. Empowering leaders optimize working conditions for engagement: a multilevel study. *J Occup Health Psychol*. 2012;17(1):15–27. doi:10.1037/a0025942 22409390

[16] Zhang X, Bartol KM. Linking empowering leadership and employee creativity: the influence of psychological empowerment, intrinsic motivation, and creative process engagement. *Acad Manage J*. 2010;53(1):107–128. doi:10.5465/amj.2010.48037118

[17] Martin SL, Liao H, Campbell EM. Directive versus empowering leadership: a field experiment comparing impacts on task proficiency and proactivity. *Acad Manage J*. 2012;56(5):1372–1395. doi:10.5465/amj.2011.0113

[18] Kim M, Beehr TA, Prewett MS. Employee responses to empowering leadership: a meta-analysis. *J Leadersh Organ Stud*. 2018;25(3):257–276. doi:10.1177/1548051817750538

[19] Kundu S, Kumar S, Gahlawat N. Empowering leadership and job performance: mediating role of psychological empowerment. *Manage Res Rev*. 2019;42(5):605–624. doi:10.1108/MRR-04-2018-0183

[20] Lee A, Willis S, Tian AW. Empowering leadership: a meta‐analytic examination of incremental contribution, mediation, and moderation. *J Organ Behav*. 2017;39(3):249–383.

[21] Zhang S, Ke X, Wang X-HF, Liu J. Empowering leadership and employee creativity: a dual-mechanism perspective. *J Occup Organ Psychol*. 2018;91(4):896–917. doi:10.1111/joop.12219

[22] Kim D, Moon CW, Shin J. Linkages between empowering leadership and subjective well-being and work performance via perceived organizational and co-worker support. *Leadersh Organ Dev J*. 2018;39(7):844–858. doi:10.1108/LODJ-06-2017-0173

[23] Hansbrough T, Jones G. Inside the minds of narcissists: how narcissistic leaders’ cognitive processes contribute to abusive supervision. *Z Psychol*. 2014;222(4):214–220.

[24] May D, Wesche J, Heinitz K, Kerschreiter R. Coping with destructive leadership: putting forward an integrated theoretical framework for the interaction process between leaders and followers. *Z Psychol*. 2014;222(4):203–213.

[25] Steele JP. Antecedents and consequences of toxic leadership in the U.S. Army: a two-year review and recommended solutions (Technical Report 2011-3). UK: Center for Army Leadership; 2011. Available from: https://apps.dtic.mil/dtic/tr/fulltext/u2/a545383.pdf. Accessed September 28, 2021.

[26] Green JE. Toxic leadership in educational organizations. *Educ Leadersh Rev*. 2014;15(1):18–33.

[27] Goldman A, Goldman A. High toxicity leadership: borderline personality disorder and the dysfunctional organization. *J Manage Psychol*. 2006;21(8):733–746. doi:10.1108/02683940610713262

[28] Lipman-Blumen J. The allure of toxic leaders: why followers rarely escape their clutches. *Ivey Bus J*. 2005;69(3):1–8.

[29] Whicker ML. *Toxic Leaders: When Organizations Go Bad*. Westport, Conn.: Quorum Books; 1996.

[30] Reed G. Toxic leadership. Military Review; 2004:67–71. Available from: http://www.george-reed.com/uploads/3/4/4/5/34450740/toxic_leadership.pdf. Accessed September 23, 2021.

[31] Ortega A. *Academic Libraries and Toxic Leadership*. Cambridge MA: Chandos Publishing; 2017.

[32] Lipman-Blumen J. Toxic leadership: when grand illusions masquerade as noble visions. *Leader to Leader*. 2005;2005(36):29–36. doi:10.1002/ltl.125

[33] Walton M. Leadership toxicity – an inevitable affliction of organizations? *Organ People*. 2007;14(1):19–27.

[34] Milosevic I, Maric S, Lončar D. Defeating the toxic boss: the nature of toxic leadership and the role of followers. *J Leadersh Organ Stud*. 2020;27(2):117–137. doi:10.1177/1548051819833374

[35] Chua SMY, Murray DW. How toxic leaders are perceived: gender and information-processing. *Leadersh Organ Dev J*. 2015;36(3):292–307. doi:10.1108/LODJ-06-2013-0076

[36] Bhandarker A, Rai S. Toxic leadership: emotional distress and coping strategy. *Int J Organ Theory Behav*. 2019;22(1):65–78. doi:10.1108/IJOTB-03-2018-0027

[37] Williams KR. Toxic leadership in defense and federal workplaces: sabotaging the mission and innovation. *Int J Public Leadersh*. 2018;14(3):179–198. doi:10.1108/IJPL-04-2018-0023

[38] Webster V, Brough P, Daly K. Fight, flight or freeze: common responses for follower coping with toxic leadership. *Stress Health*. 2014;32(4):346–354. doi:10.1002/smi.2626 25470138

[39] Mehta S, Maheshwari GC. Consequence of toxic leadership on employee job satisfaction and organizational commitment. *J Contemp Manage Res*. 2013;8(2):1–23.

[40] Omar AT, Robinson S, Dudau A. Leaders behaving badly: constructing a toxic leadership for public university leaders. *Int J Manage Appl Sci*. 2017;3(6):75–80.

[41] Wilson-Starks KY. Toxic leadership. Transleadership, Inc.; 2003. Available from: https://transleadership.com/wp-content/uploads/ToxicLeadership.pdf. Accessed September 28, 2021.

[42] Baron RA. Motivation in work settings: reflections on the core of organizational research. *Motiv Emot*. 1991;15(1):1–8. doi:10.1007/BF00991472

[43] Lam CF, Gurland ST. Self-determined work motivation predicts job outcomes, but what predicts self-determined work motivation? *J Res Pers*. 2008;42(4):1109–1115. doi:10.1016/j.jrp.2008.02.002

[44] Tremblay MA, Blanchard CM, Taylor S, Pelletier LG, Villeneuve M. Work extrinsic and intrinsic motivation scale: its value for organizational psychology research. *Can J Behav Science*. 2009;41(4):213–226. doi:10.1037/a0015167

[45] Deci EL. The history of motivation in psychology and its relevance for management. In: Vroom VH, Deci EL, editors. *Management and Motivation*. 2nd ed. London: Penguin; 1992:9–29.

[46] Ryan RM, Deci EL. Self-determination theory and the facilitation of intrinsic motivation, social development, and wellbeing. *Am Psychol*. 2000;55(1):68–78. doi:10.1037/0003-066X.55.1.68 11392867

[47] Vroom VH. *Work and Motivation*. New York: Wiley; 1964.

[48] Gagné M, Forest J, Vansteenkiste M, et al. The Multidimensional Work Motivation Scale: validation evidence in seven languages and nine countries. *Eur J Work Organ Psychol*. 2015;24(2):178–196. doi:10.1080/1359432X.2013.877892

[49] Van den Broeck A, Schreurs B, De Witte H, Vansteenkiste M, Germeys F, Schaufeli W. Understanding workaholics’ motivations: a self-determination perspective. *Appl Psychol*. 2001;60(4):600–621. doi:10.1111/j.1464-0597.2011.00449.x

[50] Gagné M, Deci EL. Self-determination theory and work motivation. *J Organ Behav*. 2005;26:331–362. doi:10.1002/job.322

[51] Grant AM, Berry JW. The necessity of others is the mother of invention: intrinsic and prosocial motivations, perspective-taking, and creativity. *Acad Manage J*. 2011;54(1):73–96. doi:10.5465/amj.2011.59215085

[52] Hetland H, Hetland J, Andreassen CS, Pallesen S, Notelaers G. Leadership and fulfillment of the three basic psychological needs at work. *Career Dev Int*. 2011;16(5):507–523. doi:10.1108/13620431111168903

[53] Kovjanic S, Schuh SC, Jonas K, Van Quaquebeke N, Van Dick R. How do transformational leaders foster positive employee outcomes? A self-determination-based analysis of employees’ needs as mediating links. *J Organ Behav*. 2012;33(8):1031–1052. doi:10.1002/job.1771

[54] Silva MN, Marques MM, Teixeira PJ. Testing theory in practice: the example of self-determination theory-based interventions. *Eur Health Psychol*. 2014;16(5):171–180.

[55] Deci EL, Olafsen AH, Ryan RM. Self-determination theory in work organizations: the state of a science. *Annu Rev Organ Psychol Organ Behav*. 2017;4:19–43. doi:10.1146/annurev-orgpsych-032516-113108

[56] Olafsen AH, Deci EL. Self-determination theory and its relation to organizations. In: *Oxford Research Encyclopedia of Psychology*. Oxford: Oxford University Press; 2020. doi:10.1093/acrefore/9780190236557.013.112

[57] Gagné M, Koestner R. Self-determination theory as a framework for understanding organizational commitment. Paper presented at: Annual Meeting of the Society for Industrial and Organizational Psychology; 2002; Toronto, Canada.

[58] Richer SF, Blanchard C, Vallerand RJ. A motivational model of work turnover. *J Appl Soc Psychol*. 2002;32(10):2089–2113. doi:10.1111/j.1559-1816.2002.tb02065.x

[59] Otis N, Pelletier LG. A motivational model of daily hassles, physical symptoms, and future work intentions among police officers. *J Appl Soc Psychol*. 2005;35(10):2193–2214. doi:10.1111/j.1559-1816.2005.tb02215.x

[60] Gagné M, Senécal CB, Koestner R. Proximal job characteristics, feelings of empowerment, and intrinsic motivation: a multidimensional model. *J Appl Soc Psychol*. 1997;27(14):1222–1240. doi:10.1111/j.1559-1816.1997.tb01803.x

[61] Miniotaitė A, Bučiūnienė I. Explaining authentic leadership work outcomes from the perspective of self determination theory. *Organizaciju vadyba*. 2013;65(11):63–75.

[62] Syahrul K. The effect of empowering leadership on intrinsic motivation: the role of psychological empowerment as a mediation. *J Leadersh Organ*. 2020;2(2):108–120.

[63] Chen G, Sharma PN, Edinger SK, Shapiro DL, Farh JL. Motivating and demotivating forces in teams: cross-level influences of empowering leadership and relationship conflict. *J Appl Psychol*. 2011;96(3):541–557. doi:10.1037/a0021886 21171730

[64] Arnold JA, Arad S, Rhoades JA, Drasgow F. The Empowering Leadership Questionnaire: the construction and validation of a new scale for measuring leader behaviors. *J Organ Behav*. 2000;21:249–269.

[65] Dos Santos NR, Mónico L, Pais L, et al. Multidimensional Work Motivation Scale: psychometric studies in Portugal and Brazil. *Manage Res*. 2022;20:89–110. doi:10.1108/MRJIAM-07-2021-1206

[66] Hair JF, Anderson RE, Tatham RL, Black WC. *Multivariate Data Analysis*. 7th ed. Harlow, Essex: Pearson Prentice-Hall; 2008.

[67] Bagozzi R, Yi Y. On the evaluation of structural equation models. *JAMS*. 1988;16(1):74–94. doi:10.1007/BF02723327

[68] Fornell C, Larcker D. Evaluating structural equation models with unobservable variables and measurement error. *J Mark Res*. 1981;18(1):39–50. doi:10.1177/002224378101800104

[69] Tabachnick BG, Fidell LS. *Using Multivariate Statistics*. 6th ed. New York: Pearson Education; 2013.

[70] Nunnally JC, Bernstein IH. *Psychometric Theory*. 3rd ed. New York: McGraw-Hill; 1994.

[71] Urbina S. *Essentials of Psychological Testing*. 2nd ed. Hoboken, New Jersey: Wiley; 2014.

[72] Schumacker RE, Lomax RG. *A Beginner’s Guide to Structural Equation Modeling*. New York: Routledge Academic; 2012.

[73] Cohen J. *Statistical Power Analysis for the Behavioural Sciences*. 2nd ed. Hillsdale, New Jersey: Erlbaum; 1988.

[74] Bacher J, Wenzig K, Vogler M. SPSS two cluster: a first evaluation. Universität Erlangen-Nurnberg; 2004. Available from: https://nbn-resolving.org/urn:nbn:de:0168-ssoar-327153. Accessed September 28, 2021.

[75] Goleman D. *Working with Emotional Intelligence*. New York: Bantam Books; 1998.

[76] Goleman D. Leadership that gets results. *Harv Bus Rev*. 2000;78(2):78–90.

[77] Schilling J, Schyns B. The causes and consequences of bad leadership [Editorial]. *Z Psychol*. 2014;222(4):187–189.

[78] Hadadian Z, Zarei J. Relation between toxic leadership and job stress of knowledge workers. *Stud Bus Econ*. 2016;11(3):84–89. doi:10.1515/sbe-2016-0037

[79] Kohut H. *The Analysis of the Self: A Systematic Approach to the Psychoanalytic Treatment of Narcissistic Personality Disorders*. New York: International Universities Press; 1971.

[80] Kets de Vries MFR. *Leaders, Fools and Imposters: Essays on the Psychology of Leadership*. San Francisco: Jossey-Bass Publishers; 1993.

[81] Maslow AH. *Eupsychian Management*. Homewood, Ill.: Irwin-Dorsey Press; 1965.

[82] Padilla A, Hogan R, Kaiser RB. The toxic triangle: destructive leaders, susceptible followers, and conducive environments. *Leadersh Q*. 2007;18(3):176–194. doi:10.1016/j.leaqua.2007.03.001

